# *Chlorella* for protein and biofuels: from strain selection to outdoor cultivation in a Green Wall Panel photobioreactor

**DOI:** 10.1186/1754-6834-7-84

**Published:** 2014-06-07

**Authors:** Alessia Guccione, Natascia Biondi, Giacomo Sampietro, Liliana Rodolfi, Niccolò Bassi, Mario R Tredici

**Affiliations:** 1 Dipartimento di Scienze delle Produzioni Agroalimentari e dell’Ambiente - Sezione di Microbiologia Agraria, Università degli Studi di Firenze, Piazzale delle Cascine 24, Firenze 50144, Italy; 2 Fotosintetica & Microbiologica S.r.l., Via dei Della Robbia 54, Firenze 50132, Italy

**Keywords:** *Chlorella*, Outdoor cultivation, Food, Thermotolerance, Nitrogen starvation, Sustainability, GWP-II, Biofuel

## Abstract

**Background:**

*Chlorella* is one of the few microalgae employed for human consumption. It typically has a high protein content, but it can also accumulate high amounts of lipids or carbohydrates under stress conditions and, for this reason, it is of interest in the production of biofuels. High production costs and energy consumption are associated with its cultivation. This work describes a strategy to reduce costs and environmental impact of *Chlorella* biomass production for food, biofuels and other applications.

**Results:**

The growth of four *Chlorella* strains, selected after a laboratory screening, was investigated outdoors in a low-cost 0.25 m^2^ GWP-II photobioreactor. The capacity of the selected strains to grow at high temperature was tested. On the basis of these results, in the nitrogen starvation trials the culture was cooled only when the temperature exceeded 40°C to allow for significant energy savings, and performed in a seawater-based medium to reduce the freshwater footprint. Under nutrient sufficiency, strain CH2 was the most productive. In all the strains, nitrogen starvation strongly reduced productivity, depressed protein and induced accumulation of carbohydrate (about 50%) in strains F&M-M49 and IAM C-212, and lipid (40 - 45%) in strains PROD1 and CH2. Starved cultures achieved high storage product productivities: 0.12 g L^−1^ d^−1^ of lipids for CH2 and 0.19 g L^−1^ d^−1^ of carbohydrates for F&M-M49. When extrapolated to large-scale in central Italy, CH2 showed a potential productivity of 41 t ha^−1^ y^−1^ for biomass, 16 t ha^−1^ y^−1^ for protein and 11 t ha^−1^ y^−1^ for lipid under nutrient sufficiency, and 8 t ha^−1^ y^−1^ for lipid under nitrogen starvation.

**Conclusions:**

The environmental and economic sustainability of *Chlorella* production was enhanced by growing the organisms in a seawater-based medium, so as not to compete with crops for freshwater, and at high temperatures, so as to reduce energy consumption for cooling. All the four selected strains are good candidates for food or biofuels production in lands unsuitable for conventional agriculture. *Chlorella* strain CH2 has the potential for more than 80 tonnes of biomass, 32 tonnes of protein and 22 tonnes of lipid per year under favourable climates.

## Background

*Chlorella* (Chlorophyta, Trebouxiophyceae), one of the most studied microalgae, is commercially cultivated by more than 70 companies in the world [1]. The annual production of *Chlorella* biomass exceeds 2,000 tonnes [1,2], mostly used for dietary supplements and nutraceuticals, with a minor share destined to the cosmetic market and aquaculture [1]. *Chlorella* is commercially produced under photoautotrophic conditions, mainly in open ponds (both raceway and circular) [3,4], or heterotrophically in fermenters [4]. The largest closed system used for autotrophic production at commercial scale is the 700 m^3^ tubular photobioreactor operated by Roquette Klötze GmbH & Co. KG (Klötze, Germany), which produces annually about 100 tonnes of high quality *Chlorella* biomass for the health food market [5].

When molecular data became available it was clear that different green microalgae with similar morpho-physiological characters had been classified as ‘*Chlorella*’. The taxonomy of the genus is still under revision [6]. *C. vulgaris* and *C. pyrenoidosa* are the two most cultivated at commercial scale. The latter species still has an uncertain taxonomic collocation [6,7]. *Chlorella* thrives in fresh or brackish waters, but several marine strains are also known. In this respect, it is important to avoid confusion with the so-called ‘marine chlorella’, a much researched organism in the 1980s because of its high eicosapentaenoic acid (EPA) content, which was later correctly identified as *Nannochloropsis* sp. [8].

*Chlorella* is one of the few microalgae (together with *Dunaliella*, *Haematococcus* and *Arthrospira*) largely employed for human consumption. It has a high protein content with a balanced amino acid composition [9,10], besides a good content of vitamins, minerals, pigments [10] and short-chain polyunsaturated fatty acids, including oleic and linoleic acids [11,12]. Some strains are also a good source of lutein [13]. *Chlorella* is recognized as a safe food ingredient worldwide [14,15], mainly due to its long history of human consumption as a food supplement and nutraceutical [7,9,16,17]. *In vivo* studies on its potential as food and protein source have been carried out mainly in the past [18,19], when legislation concerning trials on people was less restrictive [18]. More recently, *Chlorella* biomass has been proposed as a food ingredient: as colouring agent for traditional butter cookies [20], as additive for fermented milk and yoghurt to enhance the viability of bacterial probiotics [21,22] and incorporated in pasta products to increase their nutritional quality [23]. A *Chlorella* protein hydrolysate has also been tested as a food additive [24]. The food and feed markets require large quantities of biomass produced at low cost (less than 1 € kg^−1^) [25]. Currently, algae production costs are higher than 4 - 5 € kg^−1^ and, although recent economic analyses foresee a decrease to 1 - 2 € kg^−1^[26], the commercialization of *Chlorella* as a food commodity is not mature yet.

High production costs are also the main limitation to another potential application of this microalga: biofuel production. In the last decade, algal biofuels have received a great deal of attention [27]. *Chlorella* is among the algae of major interest for biofuels, since under stress and depending on the strain, it can accumulate large amounts of lipids [28] or synthesize starch [29,30]. Research carried out under nitrogen or phosphorus starvation has shown significant lipid accumulation (up to about 50%) and high lipid productivities [31,32]. Studies have also been carried out under nutrient replete conditions. Moheimani cultivated *Chlorella* sp. in a 120 L bag photobioreactor, obtaining a biomass productivity during summer of up to 0.28 g L^−1^ d^−1^ and a lipid content of about 25% [33]. Přibyl *et al.*, with *Chlorella vulgaris* in a 150 L, 6.6 m^2^ thin-layer open system, obtained maximum biomass and lipid productivities of 1.26 and 0.33 g L^−1^ d^−1^, respectively [34]. Some *Chlorella* are also highly productive in starch, and thus potential substitutes of starch-rich terrestrial plants for bioethanol production. Brányiková *et al.* increased starch content of *Chlorella* up to 50% by applying sulfur limitation in an outdoor thin-layer open system [35].

The extracted (delipidated) *Chlorella* biomass, still rich in proteins, carbohydrates, minerals and bioactive compounds, could provide raw materials for feed and food applications [36,37]. This is important in view of recent analyses that have shown that to achieve a positive energy balance and produce economically viable biofuels, the residue after extraction must be used for co-products [38-40]. A different approach, which seems more practical and feasible, is targeting feed, food or chemicals as the first product. After the extraction of the valuable compound, recovery of the residual energy (and nutrients) of the spent biomass by alcoholic fermentation or anaerobic digestion could be carried out. Integrating food and fuel production processes, besides providing economic advantages, would lead to a higher environmental sustainability [41,42]. However, the issue of matching markets of different sizes, such as that of biofuels and high-value products, must be considered.

Microalgae have several advantages over traditional crops. Their cultivation does not need fertile soil and they are very efficient in using nutrients, thus avoiding or limiting pollution of water bodies by unused fertilizers. Some algae can be cultivated in brackish, saline or seawater, thus they do not compete for dwindling freshwater resources. The use of wastewaters as a nutrient source is also an attractive possibility that can be considered when biofuels are the target. Microalgae cultures can be fed with CO_2_ from flue gases [42-46]; however, the need to supply CO_2_ to the culture should be seen as a limitation, compared to plants that absorb CO_2_ directly from the air, rather than an advantage. To make microalgal biomass economically competitive and sustainable, either for food or biofuels, the cost of the culture system, as well as operational costs, must be significantly reduced [44,45]. In particular, mixing [26,44,45,47,48] and cooling [44,45,47] costs, which are very high in closed systems, need to be cut substantially by, for example, selecting strains with high buoyancy and able to grow at high temperatures [49,50]. For sustainable microalgae cultivation, strains able to grow with high productivity in seawater or brackish water are required.

The aim of this work was to evaluate the performance (in terms of protein, carbohydrate and lipid content and productivity) of selected *Chlorella* strains grown under conditions devised to reduce operational costs and increase the sustainability of the cultivation process. To reach this goal, outdoor growth experiments with four strains, selected after a thorough laboratory screening, were carried out in a low-cost photobioreactor, the Green Wall Panel (GWP), with reduced or without cooling in a seawater-based, instead of the standard freshwater-based, culture medium. Cultivation in nitrogen deprived media was finally tested to increase storage product accumulation (lipid or carbohydrate) and evaluate the potential of the selected strains for biofuel (biodiesel or ethanol) production.

## Results

### Laboratory screening of nine *Chlorella* strains

Nine *Chlorella* strains were cultivated in 300 mL bubble tubes in the laboratory to evaluate their productivity and biochemical composition in nutrient sufficient and nitrogen deprived growth media. Under nutrient sufficiency, batch and semi-continuous cultures were compared. With two exceptions (IRT2 and CH2), batch cultures achieved higher productivities (on average 0.68 versus 0.55 g L^−1^ d^−1^). The more productive batch cultures were those of strains MACH1, CH2, PROD1, IAM C-212 and PAVV2P2, all above 0.7 g L^−1^ d^−1^. In semi-continuous culture, only strain CH2 attained a high productivity (0.82 g L^−1^ d^−1^) (Table 1). Under nitrogen starvation (evaluated only in batch), the average biomass productivity decreased from 0.68 to 0.37 g L^−1^ d^−1^. The decrease, observed for all the strains, ranged from a minimum of 31% for MACH1 to a maximum of 75% for IAM C-212 (Table 1).

**Table 1:** **Productivities of nine *Chlorella *** **strains grown in laboratory conditions in 300 mL tubes in their isolation medium**

**Strain**	**Nutrient sufficient medium**		**Nitrogen starved medium**
	**Batch (g L**^**−1**^**d**^**−1**^**)**	**Semi-continuous (g L**^**−1**^**d**^**−1**^**)**	**Batch (g L**^**−1**^**d**^**−1**^**)**
F&M-M49	0.64 ± 0.05	0.54 ± 0.04	0.23 ± 0.02
CCAP 211-11b	0.59 ± 0.02	0.30 ± 0.04	0.32 ± 0.04
IAM C-212	0.71 ± 0.05	0.58 ± 0.05	0.18 ± 0.04
PROD1	0.73 ± 0.01	0.50 ± 0.05	0.43 ± 0.03
PAVV2P2	0.71 ± 0.02	0.50 ± 0.05	0.46 ± 0.03
IRT2	0.62 ± 0.00	0.63 ± 0.00	0.29 ± 0.02
BdR3	0.67 ± 0.02	0.51 ± 0.02	0.34 ± 0.04
MACH1	0.78 ± 0.02	0.58 ± 0.03	0.54 ± 0.03
CH2	0.75 ± 0.02	0.82 ± 0.07	0.50 ± 0.04

The biochemical composition of biomasses harvested at the end of the experiment from batch cultures is shown in Table 2. With the exception of one strain (BdR3), under nutrient sufficiency, biomasses showed a good protein content (about 40%). Carbohydrates varied from a minimum of 24.2% for F&M-M49 to a maximum of 35.6% for BdR3, while lipids ranged from 20.0% for PAVV2P2 to 28.1% for PROD1. Under nitrogen deprivation the protein content decreased substantially in all the strains (on average to about 25%), three strains (CCAP 211-11b, PROD1 and CH2) accumulated lipids up to more than 45% and the other six accumulated carbohydrate up to about 50%. No strain accumulated both carbohydrate and lipids. The highest lipid productivity was obtained with strains CH2 and PROD1. The highest carbohydrate productivity was attained by strains IAM C-212 and F&M-M49 (data not shown).

**Table 2:** **Biochemical composition of nine *Chlorella *** **strains grown in laboratory conditions in 300 mL tubes**

**Strain**	**Nutrient sufficient medium**				**Nitrogen starved medium**			
	**Protein (%)**	**Carbohydrate (%)**	**Lipid (%)**	**Ash (%)**	**Protein (%)**	**Carbohydrate (%)**	**Lipid (%)**	**Ash (%)**
F&M-M49	45.4 ± 1.57	24.2 ± 0.60	22.8 ± 1.89	4.8	28.2 ± 2.74	53.0 ± 0.51	15.4 ± 0.60	5.4
CCAP 211-11b	44.1 ± 1.18	26.6 ± 2.85	22.0 ± 2.13	5.6	25.2 ± 0.03	24.3 ± 3.34	46.0 ± 1.34	5.2
IAM C-212	40.5 ± 0.16	26.8 ± 0.23	24.3 ± 0.76	6.3	25.9 ± 3.76	51.9 ± 0.27	17.1 ± 0.67	6.9
PROD1	39.9 ± 0.84	28.3 ± 0.46	28.1 ± 0.20	5.2	26.8 ± 1.46	21.5 ± 3.87	47.4 ± 0.06	5.9
PAVV2P2	45.3 ± 2.15	27.1 ± 0.61	20.0 ± 1.28	5.8	22.1 ± 0.04	48.6 ± 2.65	22.5 ± 0.39	4.8
IRT2	38.0 ± 1.84	35.2 ± 0.67	20.5 ± 0.03	6.3	20.2 ± 2.82	47.1 ± 0.93	22.3 ± 0.50	8.5
BdR3	25.8 ± 3.05	35.6 ± 3.08	26.0 ± 0.04	8.3	24.6 ± 0.20	50.9 ± 2.06	17.8 ± 2.03	7.8
MACH1	39.4 ± 3.37	29.7 ± 4.84	25.6 ± 0.24	4.9	28.9 ± 4.18	47.7 ± 2.22	12.1 ± 0.07	8.2
CH2	39.3 ± 1.63	28.8 ± 0.25	23.1 ± 0.03	12.2	20.1 ± 3.79	20.3 ± 0.56	50.8 ± 1.43	12.0

A second laboratory trial was carried out with the nine *Chlorella* strains to compare growth in freshwater- and seawater-based media and test their ability to grow when the culture temperature was maintained, during the light hours, at 40°C, a value easily reached during outdoor cultivation in closed systems when cooling is not applied. Three strains (PAVV2P2, CCAP 211-11b and PROD1) did not survive exposure to these high temperatures (Table 3). Four strains (F&M-M49, IAM C-212, MACH1 and CH2) showed higher productivities when cultured for 8 hours a day at 40°C, with respect to continuous cultivation at 25°C. The best performance at high temperature was obtained by CH2 (with a productivity of 1 g L^−1^ d^−1^), followed by IAM C-212 (0.80 g L^−1^ d^−1^), MACH1 (0.69 g L^−1^ d^−1^) and F&M-M49 (0.57 g L^−1^ d^−1^). In the seawater-based F medium all the nine strains grew well, and strains CH2, isolated from seawater, PROD1 and IRT2 achieved higher productivities compared to the freshwater-based BG11 medium (Table 3).

**Table 3:** **Productivities of nine *Chlorella *** **strains at high temperature and in different culture media in laboratory conditions in 40 mL tubes**

**Strain**	**High temperature trial**		**Culture medium trial**	
	**Control 8 h:16 h**^**a**^**at 25°C**^**c**^**(g L**^**−1**^**d**^**−1**^**)**	**High temperature 8 h at 40°C; 16 h at 25°C**^**c**^**(g L**^**−1**^**d**^**−1**^**)**	**Freshwater medium 24 h**^**b**^**; 25°Cconstant (g L**^**−1**^**d**^**−1**^**)**	**Seawater medium 24 h**^**b**^**; 25°Cconstant (g L**^**−1**^**d**^**−1**^**)**
F&M-M49	0.42 ± 0.02	0.57 ± 0.01	0.37 ± 0.02	0.33 ± 0.00
CCAP211-11b	0.34 ± 0.02	NG	0.56 ± 0.01	0.42 ± 0.01
IAM C-212	0.66 ± 0.02	0.80 ± 0.05	0.40 ± 0.01	0.43 ± 0.01
PROD1	0.64 ± 0.01	NG	0.53 ± 0.02	0.73 ± 0.01
PAVV2P2	0.30 ± 0.01	NG	0.42 ± 0.01	0.38 ± 0.02
IRT2	0.59 ± 0.02	0.41 ± 0.02	0.48 ± 0.01	0.66 ± 0.01
BdR3	0.34 ± 0.01	0.16 ± 0.02	0.58 ± 0.00	0.60 ± 0.02
MACH1	0.27 ± 0.02	0.69 ± 0.02	0.30 ± 0.01	0.24 ± 0.00
CH2	0.45 ± 0.01	1.00 ± 0.04	0.44 ± 0.01	0.58 ± 0.01

^a^8 h:16 h, light:dark cycle; ^b^24 h, continuous illumination; ^c^culture performed in each strain isolation medium; NG, no growth.

From the laboratory screening four *Chlorella* strains (IAM C-212, PROD1, F&M-M49 and CH2) emerged as promising for sustainable biomass and energy feedstock production, and were selected for further study. The main selection criteria were productivity, capacity to grow in a seawater-based medium and at high temperatures, and type of storage product accumulated under nitrogen starvation. The four selected strains were evaluated outdoors, first in 1 L bubble tubes and then in 0.25 m^2^ GWP-II reactors.

### Outdoor cultivation of four selected *Chlorella* strains without temperature control in bubble tubes and in 0.25 m^2^ GWP-II reactors

The four *Chlorella* strains were cultivated outdoors in 1 L bubble tubes in either BG11 or F medium in a water bath without temperature control. The temperature stayed above 35°C for an average of about 1.5 hours a day, but never exceeded 38°C. The average global solar radiation during the experimental period was 27.4 MJ m^−2^ d^−1^. Higher productivities were always achieved in F medium (Table 4) irrespective of the habitat of origin of the strains. In the case of PROD1, productivity in the seawater-based medium almost tripled with respect to the freshwater-based medium. Strain CH2 in the seawater-based medium achieved 1 g L^−1^ d^−1^ (Table 4).

**Table 4:** **Productivity of four selected *Chlorella *** **strains cultivated outdoors in 1 L tubes without temperature control**

**Strain**	**BG11 (g L**^**−1**^**d**^**−1**^**)**	**F (g L**^**−1**^**d**^**−1**^**)**
F&M-M49	0.36 ± 0.03	0.69 ± 0.01
IAM C-212	0.33 ± 0.01	0.48 ± 0.01
PROD1	0.23 ± 0.08	0.63 ± 0.02
CH2	0.87 ± 0.23	1.02 ± 0.01

Given the results of the previous outdoor experiment in tubes, the four selected strains were grown in north-south oriented 0.25 m^2^ vertical GWP-II reactors without temperature control. When the culture temperature stayed above 40°C for 3 - 4 hours every day, none of the cultures survived more than 3 days neither in BG11 nor in F medium (data not shown). Besides, the protein content was always low (21 to 26%). Carbohydrates in F&M-M49 (47%) and IAM C-212 (44%) or lipids in CH2 (34%) and PROD1 (32%) accumulated well beyond the typical content.

### Outdoor cultivation of four selected *Chlorella* strains in 0.25 m^2^ GWP-II reactors in nutrient sufficient and nitrogen deprived media

**Figure 1:**
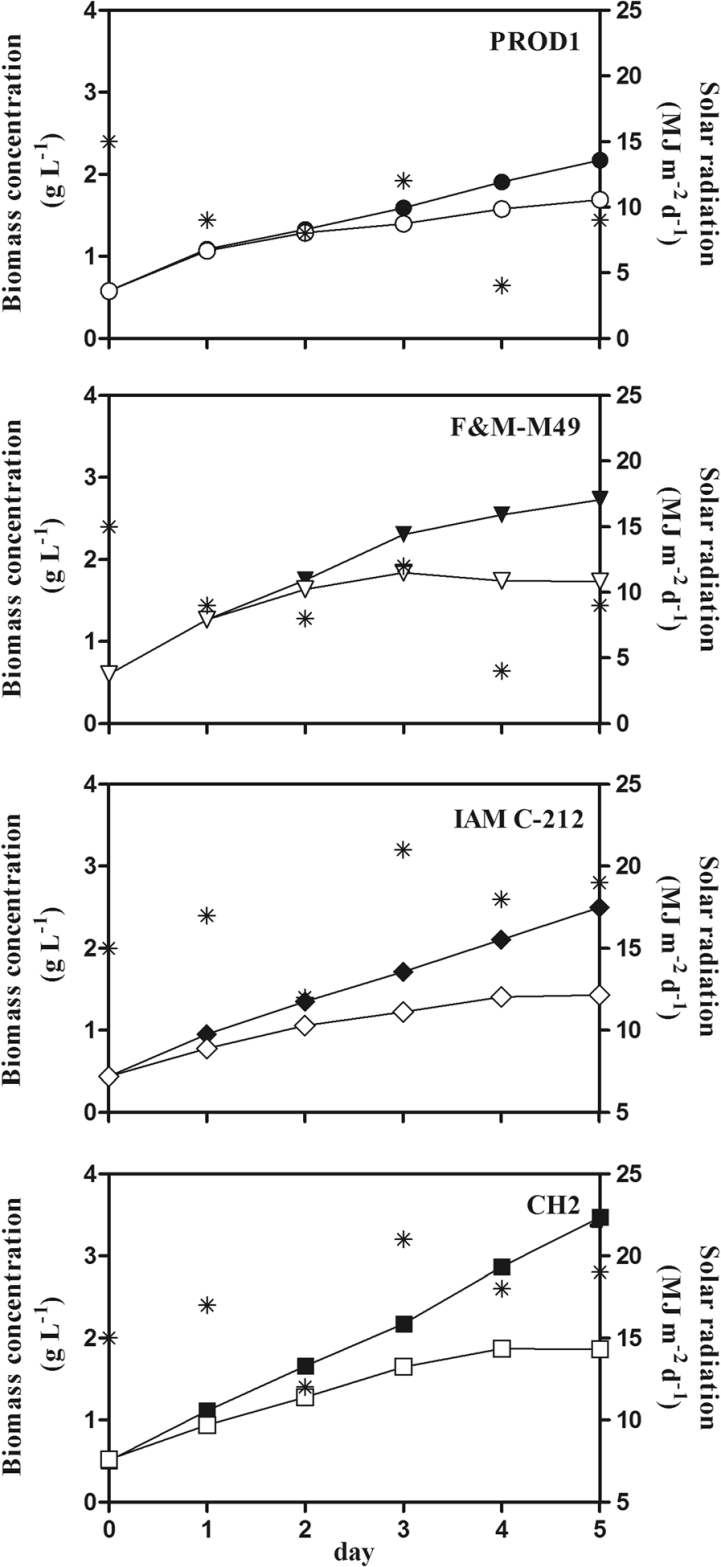
**Growth of four selected *Chlorella *strains cultivated outdoors in 0.25 m**^**2 **^**GWP-II under nitrogen starvation and nutrient sufficiency.** Growth, expressed as biomass concentration, of four *Chlorella* strains in 0.25 m^2^ GWP-II reactors in F medium. Cultures under nutrient sufficiency are indicated by filled symbols, starved cultures by empty symbols. Daily global solar radiation is indicated by an asterisk. Temperature controlled at 40°C.

The four selected strains were grown in north-south oriented 0.25 m^2^ vertical GWP-II reactors to compare performances in nitrogen sufficient and nitrogen starved seawater-based media. Given the negative results of the previous outdoor trial without temperature control, for this trial the control system was set up so as to allow the culture temperature to increase during daylight but never surpass 40°C. The nutrient sufficient cultures grew well for the 5 days of the trial (Figure 1). CH2 was by far the most productive (0.6 g L^−1^ d^−1^) (Table 5). Under nitrogen starvation F&M-M49 ceased to grow after 3 days and IAM C-212 and CH2 after 4 days (Figure 1). Only PROD1 grew, albeit slowly, for the whole experimental period. Nitrogen starvation strongly reduced productivity in all the strains: of about 50% in F&M-M49, IAM C-212 and CH2, and of 30% in PROD1 (Table 5). In nutrient sufficient conditions biomass composition was similar in all the strains, with a protein content of about 40% and an about half content of carbohydrate (20 to 25%) and lipid (20 to 26%), thus confirming previous laboratory and outdoor results. Nitrogen starvation strongly reduced protein in all the strains, and triggered a gradual accumulation of the typical storage product, which reached the maximum content after 5 days. As observed in the laboratory, F&M-M49 and IAM C-212 accumulated carbohydrate (up to about 50%), while PROD1 and CH2 accumulated lipids (up to about 40%) (Table 6). The high lipid productivity of CH2 (0.12 g L^−1^ d^−1^) and, particularly, the high carbohydrate productivity of F&M-M49 (0.19 g L^−1^ d^−1^) under nitrogen starvation were noteworthy (Table 5). However, lipid productivity in CH2 was higher in nutrient sufficiency.

**Table 5:** **Productivity of four selected *Chlorella *** **strains cultivated outdoors in 0.25 m**^**2 **^**GWP-II under nitrogen starvation and nutrient sufficiency**

**Strain**	**Nutrient sufficient cultures**		**Starved cultures**	
	**Biomass (g L**^**−1**^**d**^**−1**^**)**	**Storage product (g L**^**−1**^**d**^**−1**^**)**	**Biomass (g L**^**−1**^**d**^**−1**^**)**	**Storage product (g L**^**−1**^**d**^**−1**^**)**
F&M-M49	0.43	0.08 carbohydrate	0.23	0.19 carbohydrate
IAM C-212	0.41	0.14 carbohydrate	0.20	0.12 carbohydrate
PROD1	0.32	0.06 lipid	0.22	0.10 lipid
CH2	0.60	0.16 lipid	0.27	0.12 lipid

**Table 6:** **Biomass biochemical composition of four selected *Chlorella *** **strains cultivated outdoors in 0.25 m**^**2 **^**GWP-II under nitrogen starvation and nutrient sufficiency**

**Strain**	**Nutrient sufficient cultures**				**Nitrogen starved cultures**			
	**Protein (%)**	**Carbohydrate (%)**	**Lipid (%)**	**Ash (%)**	**Protein (%)**	**Carbohydrate (%)**	**Lipid (%)**	**Ash (%)**
F&M-M49	40.5 ± 4.38	20.3 ± 2.04	19.7 ± 0.22	17.4	17.5 ± 3.88	46.6 ± 0.66	16.1 ± 0.56	16.6
IAM C-212	39.8 ± 4.38	24.6 ± 0.57	24.2 ± 0.02	11.5	16.6 ± 3.28	51.0 ± 0.68	18.4 ± 0.05	13.3
PROD1	41.3 ± 2.82	25.4 ± 0.61	20.2 ± 0.16	13.0	25.7 ± 2.78	22.2 ± 1.45	39.2 ± 0.03	13.2
CH2	38.9 ± 3.67	20.8 ± 1.30	26.0 ± 0.02	15.1	26.1 ± 3.32	17.2 ± 0.59	40.0 ± 0.15	13.8

During this experiment, carried out in September, with an average solar radiation on the horizontal of 17.0 MJ m^−2^ d^−1^, the 0.25 m^2^ vertical panel intercepted on average 4.26 MJ d^−1^. Under nutrient sufficiency strain CH2 produced 6 g biomass d^−1^, 2.4 g protein d^−1^ and 1.6 g lipid d^−1^. Under nitrogen starvation lipid production decreased to 1.2 g d^−1^. The photosynthetic efficiency (PE) achieved by strain CH2 was thus: 1.4 g biomass, 0.55 g protein and 0.38 g lipid per MJ received, under nutrient sufficiency, and 0.28 g lipid per MJ received under nitrogen starvation.

### Biomass and lipid productivity potential of *Chlorella* strain CH2

From the biomass, lipid and protein productivity data attained with *Chlorella* strain CH2 in the isolated vertical GWP in September, we extrapolated the areal productivity of a scaled-up (1 ha) plant of panels. The rationale for the calculation was the following: in the 1 ha plant the GWP reactors are placed in south-facing parallel rows at the minimum distance (D) between rows that avoids shading of direct radiation among the panels. This distance varies monthly and allows the scaled-up panels to receive the same amount of direct sunlight as an isolated single row of panels. Diffuse light received by the scaled-up panels will be, on the contrary, reduced by a significant fraction compared to the single row because they are closely spaced and see only a portion of the sky. The diffuse and ground-reflected radiation on the panels was calculated for each month from April to September and added to the intercepted direct radiation to have the total radiation impinging on the panels. The monthly total radiation on the scaled-up panels and the PE values obtained in our experiments by strain CH2 (see previous paragraph) were finally used to calculate the potential productivity (in terms of biomass, protein and lipid) of the 1 ha plant (Table 7).

**Table 7:** **Biomass, lipid and protein potential productivity of *Chlorella *** **strain CH2 in a 1 ha GWP-II plant located in central Italy**

**Month**	**Average total solar radiation on the horizontal (GJ ha**^**−1**^ **month**^**−1**^**)**^**a**^	**Minimum distance D (m)**	**Total radiation on panels (GJ ha**^**−1**^ **month**^**−1**^**)**	**Biomass productivity (t ha**^**−1**^ **month**^**−1**^**)**	**Protein productivity (t ha**^**−1**^ **month**^**−1**^**)**	**Lipid productivity (t ha**^**−1**^ **month**^**−1**^**) + N/-N**
April	5,280	0.40	3,590	5.06	1.97	1.32/1.00
May	6,975	0.26	4,883	6.88	2.68	1.79/1.37
June	7,410	0.20	5,261	7.42	2.89	1.93/1.47
July	8,060	0.20	5,884	8.30	3.24	2.16/1.65
August	6,540	0.25	5,569	7.85	3.06	2.04/1.56
September	4,950	0.40	3,960	5.58	2.18	1.45/1.11

^a^Data retrieved from the Photovoltaic Geographical Information System (PVGIS) (http://re.jrc.ec.europa.eu/pvgis) for Florence latitude; D, minimum distance that avoids shading; +N, under nutrient sufficiency; −N, nitrogen starvation.

## Discussion

This study was aimed to find a strategy to reduce the costs and environmental impacts of *Chlorella* biomass production under autotrophic conditions in large-scale plants. In particular, the following changes with respect to the standard industrial cultivation procedures were investigated: the use of a seawater-based medium, none or limited temperature control, nitrogen deprivation when carbohydrates or lipids are the target products. To this end, nine *Chlorella* strains were first screened in the laboratory to test their performance under different culture conditions. Then the four best strains were evaluated outdoors.

Temperature control (cooling) is one of the main operative costs of algal biomass production in closed reactors [45,51]. To reduce costs related to thermoregulation, the use of thermotolerant strains has been proposed [52]. In this work, all the four selected *Chlorella* strains showed good growth at or below 38°C, when cultivated outdoors in bubble tubes, confirming the results previously obtained in the laboratory. The trials performed in GWP-II reactors showed that the four *Chlorella* strains could survive for a few days when the temperature exceeded 40°C for 3 - 4 hours a day. Few works deal with *Chlorella* cultivation at temperatures higher than 40°C, particularly outdoors. de-Bashan *et al.* showed that immobilized *C. sorokiniana* cells were able to grow when exposed for 5 hours daily at temperatures of 40°C and high irradiance (2,500 μmol photons m^−2^ s^−1^) in a 1 L fermenter [53]. Morita *et al.* cultivated *C. sorokiniana* indoors in a conical helical tubular photobioreactor covering an area of 0.5 m^2^, in which the strain was kept at 40°C and under a light intensity of 980 μmol photons m^−2^ s^−1^ (12 h:12 h light:dark cycle), obtaining a biomass productivity of 1.23 g L^−1^ d^−1^ (34.4 g m^−2^ of installation area d^−1^) [54]. Feng *et al.* studied the feasibility of *C. zofingiensis* outdoor cultivation in 60 L, 17 cm thick, flat plate photobioreactors in spring, without temperature control, obtaining maximum biomass productivities of 0.06 g L^−1^ d^−1^ (about 10 g m^−2^ of directly illuminated reactor surface d^−1^), although, in this study, the culture temperature never surpassed 40°C [31]. Béchet *et al.* cultivated *C. sorokiniana* outdoors without temperature control (maximum temperature of the culture was 41°C) in a 51 L cylinder photobioreactor, with a productivity of 0.21 g L^−1^ d^−1^ (10 g m^−2^ of directly illuminated surface d^−1^) [50]. The productivities measured by us in the GWP-II are comparable or higher than those attained in these studies carried out outdoors without temperature control. The biomass of cultures grown at inhibiting temperatures (maxima from 47 to 50°C) showed an increased content of storage product and a marked decrease of protein in all the strains. A similar change in biomass composition caused by high temperatures was shown by Han *et al.* in *C. pyrenoidosa* cultivated outdoors in a 50 L open tank; at daytime temperatures varying from 30 to 36°C, lipid content increased by about 40% compared to the culture kept below 30°C [55].

An important limitation of large-scale algae cultures for protein or energy production is competition for freshwater with traditional crops. To avoid or reduce the impact on freshwater resources, algae must be cultivated in brackish water or seawater [27,44]. No data are available, to our knowledge, on cultivation at high salinities (≥30 g L^−1^) of *Chlorella* strains isolated from freshwater. In this work, all the strains were tested for their ability to grow in a seawater-based medium. Surprisingly, outdoors without temperature control, the four selected strains performed better in the seawater-based medium independently of their habitat of origin, and thus this medium was adopted for the starvation trials in the GWP-II reactors. It is worth noting that the salinity of the growth medium did not influence biomass composition neither in nutrient sufficiency nor in nitrogen starved conditions.

In several algae, nutritional stresses, for example, deprivation of nitrogen or phosphorus, limit cell growth, while increasing lipid [43,56-58] or carbohydrate [35,59,60] content. For this reason, nutrient deficiency has been regarded as one of the most efficient approaches to increase storage product content for biofuels [43,61]. In our trials outdoors, nitrogen starvation led to a significant decrease of biomass productivity compared to nutrient sufficient conditions (on average 0.23 and 0.44 g L^−1^ d^−1^, respectively), and increased accumulation of the storage product for all the strains. Under nitrogen starvation strain F&M-M49 was the best performer in terms of carbohydrate productivity (0.19 g L^−1^ d^−1^), while CH2 was the best lipid producer (0.12 g L^−1^ d^−1^). It is also noteworthy that in the latter strain lipid productivity under nutrient sufficiency was higher than following nitrogen starvation. However, neutral lipids, suitable for biodiesel, are only or mainly accumulated under nutrient stress [56,61-63]. In all the strains, protein content decreased under nitrogen starvation; however, strains CH2 and PROD1 maintained a good protein content (about 26%) at the end of the starvation period. Given the high biomass, protein and lipid productivity in a seawater-based medium, and higher thermotolerance, strain CH2 shows high potential for food and biofuel production in hot arid climates.

Although a vast amount of literature on *Chlorella* cultivation for biofuels is now available [60,64-67], only a few works have been carried out outdoors under autotrophic conditions. Zhou *et al.* cultivated *Chlorella* sp. outdoors in a 70 L, 22 cm wide, 185 cm high vertical photobioreactor, obtaining a 47% increase in lipid content and a lipid productivity of about 15 mg L^−1^ d^−1^ under nitrogen starvation [68]. Feng *et al.* cultivated *C. zofingiensis* in a 60 L flat plate photobioreactor under nitrogen starvation attaining a maximum lipid productivity of 22.3 mg L^−1^ d^−1^ (about 4 g m^−2^ of directly illuminated reactor surface d^−1^) [31]. Münkel *et al.* cultivated *C. vulgaris* in a 30 L, 3 cm thick Flat Panel Airlift reactor under nitrogen and phosphorus starvation (14 days) [32]. The best average biomass and fatty acid productivities (about 0.67 and 0.39 g L^−1^ d^−1^ corresponding to 20.1 and 11.7 g m^−2^ of directly illuminated reactor surface d^−1^, respectively) were obtained at the highest cell concentration (4 g L^−1^). This latter productivity is among the highest ever reported. The productivity obtained by us with *Chlorella* strain CH2 grown in a seawater-based medium, both in terms of volumetric and areal productivities (0.12 g L^−1^ d^−1^ corresponding to 4.9 g m^−2^ of directly illuminated reactor surface d^−1^), compares well with the literature values. Few works are available on starch accumulation in *Chlorella* cultivated outdoors. Brányiková *et al.* cultivated *Chlorella* sp. in a thin-layer cascade pond to maximize starch content through sulfur limitation, attaining values of about 50% [35]. According to these authors, sulfur limitation increases starch to a level that would be viable for bioethanol production. Our strains F&M-M49 and IAM C-212 were able to accumulate comparable amounts of carbohydrates under nitrogen starvation.

To produce algal biomass at low cost, compatible with biofuel or food production, it is necessary to significantly reduce capital and operational costs of the culture system. In this work, the strategies used to reduce the costs of *Chlorella* production were the use of a low-cost photobioreactor, the GWP-II [44,45,51,69], and the reduction of cooling needs. In fact, all the outdoor trials were carried out with cooling activated only above 40°C. Bassi and Tredici (unpublished) have calculated that, in a plant made of east-west oriented vertical GWP-II, located in central Italy, if the culture temperature is maintained at 27°C, the energy consumption due to cooling is about 15% of the total energy used to operate the plant. Maintaining the culture temperature at 40°C reduces the cooling contribution to 1% of the total operational energy costs.

Although most of the scientific community and industry consider open ponds as more economically convenient for algae cultivation compared to photobioreactors [46,70,71], it is undeniable that ponds suffer several limitations that make them unsuitable for large-scale production of algae unless the cultivated organisms have very specific requirements (for example, high pH or high salinity). According to Norsker *et al.* production of algae in flat panels and tubular photobioreactors may become cheaper than in raceway ponds within 10 years [26].

Our work shows that significant savings can indeed be attained with thermotolerant algae and that the potential of selected *Chlorella* strains in flat reactors is very high when compared with the typical yields of traditional crops.

The potential in productivity terms of a large-scale GWP-II plant located in central Italy with *Chlorella* CH2 is of 41 tonnes of biomass, 16 tonnes of protein and 11 tonnes of lipid under nutrient sufficiency, and of 8 tonnes of lipid under nitrogen starvation. In a more favourable location (for example, North Africa) allowing year-round cultivation, the potential productivity would surpass 80, 32 and 22 tonnes per year for biomass, protein and lipid, respectively. Under nitrogen deficiency, lipid productivity decreases, but mainly neutral lipids, more suitable for biofuels, are accumulated [62] and the lipid content of the biomass rises to 40%, which greatly favors extraction. Besides, another important advantage is obtained as the amount of nutrients (for example, nitrogen) supplied to the culture can be significantly reduced.

Note that the above figures show the potential in terms of productivity, but do not provide any evaluation in terms of plant cost or energy efficiency. In reality, placing the panel rows so close to each other has benefits as it maximizes solar radiation interception, but also drawbacks in terms of high capital and operational costs. Besides, it is unlikely that a plant will adopt movable panels or erect a variable number of rows during the cultivation season. A possible solution to capture a high fraction of the solar radiation impinging on the horizontal with a more economic and simple arrangement would be to adopt a fixed minimum distance for the whole period and tilt the reactors (for example, at 45° with respect to the horizontal). This will allow a higher amount of solar energy to be harvested with a reduced number of panels. However, in this case a reduction of the PE is likely, since the beneficial effects of light dilution are lost.

Only detailed economical and life-cycle analyses will provide the solution to optimize panel orientation and placement for maximum economic return and reduced environmental impact.

## Conclusions

This work aimed to develop strategies for enhancing the environmental and economic sustainability of microalgal biomass production for food, biofuels and other feedstocks. Four selected *Chlorella* strains were cultivated outdoors in closed reactors (the GWP) with limited temperature control, so allowing for a significant saving in energy expenditure for cooling. Besides, all the strains performed very well in a seawater-based culture medium, a crucial feature if we aim at an environmentally sustainable production process. These features make feasible the cultivation of *Chlorella* in regions of high year-round solar irradiation, where temperature is generally high and freshwater availability limited [72,46], using lands and waters unsuitable for conventional agriculture. In these climates the potential productivity of *Chlorella* surpasses 80 tonnes of biomass, 32 tonnes of proteins and 22 tonnes of lipids per hectare per year. Extraction of the target product from both the nutrient sufficient and nitrogen starved biomasses (for example, protein or lipid), would leave important amounts of residues that could find application as biomaterials or energy feedstocks.

## Methods

The nine *Chlorella* strains screened in the laboratory for their growth capacity in different culture conditions are listed in Table 8. The laboratory screening trials are reported in Table 9. The outdoor trials (Table 10) were performed on four selected strains.

**Table 8:** ***Chlorella *** **strains used for the experiments**

**Strain**	**Origin**	**Isolation medium**
*Chlorella* sp. F&M-M49	Fotosintetica & Microbiologica Culture Collection (Florence, Italy)	BG11
*Chlorella vulgaris* CCAP211-11b	Culture Collection of Algae and Protozoa (Argyll, UK)	BG11
*Chlorella sorokiniana* IAM C-212	Microbial Culture Collection at the National Institute for Environmental Studies (NIES) (Tsukuba, Japan)	BG11
*Chlorella* sp. PROD1	Isolated from piggery slurry (Umbria, Italy)	BG11
*Chlorella* sp. PAVV2P2	Isolated from a pig manure storage lagoon (Lombardia, Italy)	BG11
*Chlorella* sp. IRT2	Isolated from urban drainage water (Tabriz, Iran)	BG11
*Chlorella* sp. BdR3	Isolated from thermal mud (Bagno di Romagna, Italy)	BG11
*Chlorella* sp. MACH1	Isolated from a rainwater puddle (Machala, Ecuador)	BG11
*Chlorella* sp. CH2	Isolated from a diatom culture in a hatchery (Pahang, Malaysia)	F

**Table 9:** **Plan of the experiments for screening the nine *Chlorella *** **strains in the laboratory**

**Trial**	**Duration (days)**	**Culture system**	**Culture regime**	**Light regime and intensity**	**Culture medium**	**Temperature**
1. Batch growth	7	300 mL bubble tubes	Batch	Continuous light (400 μmol photons m^−2^ s^−1^)	Isolation^a^ medium	25°C
2. Semi-continuous growth	7	300 mL bubble tubes	30% daily dilution	Continuous light (400 μmol photons m^−2^ s^−1^)	Isolation^a^ medium	25°C
3. Starvation	7	300 mL bubble tubes	Batch	Continuous light (400 μmol photons m^−2^ s^−1^)	Isolation^a^ medium	25°C
4. High temperature	4	40 mL bubble tubes	Batch	Light:dark cycle (8 h:16 h) (200 μmol photons m^−2^ s^−1^)	Isolation^a^ medium	40°C for 8 h - 25°C for 16 h; control cultures 24 h at 25°C
5. Culture medium	7	40 mL bubble tubes	Batch	Continuous light (200 μmol photons m^−2^ s^−1^)	BG11 and F	25°C

^a^F medium for CH2, BG11 medium for the other strains.

**Table 10:** **Plan of the experiments for screening the four selected *Chlorella *** **strains outdoors**

**Trial**	**Duration (days)**	**Culture system**	**Culture regime**	**Light regime and intensity**	**Culture medium**	**Temperature**
6. High temperature growth in bubble tubes	4	1 L bubble tubes	Batch	Natural illumination (July)	BG11 and F	Not controlled
7. High temperature growth in GWP reactors	4	10 L, 0.25 m^2^ GWP-II	Batch	Natural illumination (July - August)	BG11 and/or F	Not controlled
8. Starvation in GWP reactors	5	10 L, 0.25 m^2^ GWP-II	Batch	Natural illumination (September)	F	Cooled when above 40°C

GWP, Green Wall Panel.

### Laboratory culture conditions

Culture media were BG11 (freshwater-based medium) [73] and F (seawater-based medium) [74]. BG11 was sterilized in an autoclave. F medium was prepared with artificial seawater (Adriatic Sea Aquarium & Equipment, Rimini, Italy) at 30 g L^−1^ salinity, autoclaved, allowed to cool and then added with sterile nutrient solutions. For BG11 medium initial NaNO_3_ and K_2_HPO_4_ concentrations of 1.5 and 0.14 g L^−1^, respectively, were used, whereas for F medium the initial concentrations were of 1.5 and 0.11 g L^−1^, respectively. When necessary nutrients were added during cultivation according to productivity to avoid limitation, considering N as 10% and P as 1% of the biomass. For all the laboratory trials, cultures were bubbled with an air/CO_2_ mixture (98/2, v/v) to provide mixing and maintain pH in the optimal range (7.5 to 8), as well as to provide carbon. All the tubes were immersed in a thermoregulated water bath to maintain a constant temperature. For all the trials the initial cell concentration was 0.2 g L^−1^. An experimental plan of the trials is shown in Table 9. The screening trials 1, 2 and 3 on the nine *Chlorella* strains were carried out in duplicate in 7-day long experiments, using 300 mL, 40 mm in diameter glass tubes, under a continuous one-side illumination of 400 μmol photons m^−2^ s^−1^ provided by fluorescent daylight lamps. A constant temperature of 25°C was maintained. Each strain was tested in its isolation medium (Table 8). The first trial was performed in batch regime while the second in semi-continuous regime (30% of the culture volume was harvested daily and replaced with fresh medium). The third laboratory trial was carried out in nitrogen starvation in batch conditions; the trial was started by inoculating the alga in the growth medium deprived of nitrogen. Growth of starved cultures was compared to that of a culture in nutrient replete medium (control). The capacity of the strains to grow at high temperature (trial 4) was tested in duplicate in 4-day long experiments, in batch regime using 40 mL, 20 mm in diameter glass tubes. A light:dark cycle (8 h:16 h) matching with the temperature cycle, that is, 40°C during the 8 light hours and 25°C during the 16 dark hours, was applied. The cultures were illuminated from two sides with 200 μmol photons m^−2^ s^−1^ provided by metal halide lamps. The strains were tested in their isolation medium. Control cultures were maintained at 25°C applying the same light:dark cycle. A 7-day trial (Table 9, trial 5) to test the effect of culture medium was performed in 40 mL bubble tubes, in batch regime, under continuous two-side illumination and at a constant temperature of 25°C. For this trial the inocula were grown in the respective isolation medium, centrifuged, washed with deionized water and centrifuged again, then the biomass was re-suspended in the experimental culture medium.

### Outdoor culture conditions

The outdoor trials on the four selected strains were carried out at the experimental area of Fotosintetica & Microbiologica S.r.l. located in Sesto Fiorentino (Florence, Italy) in the summer (July to September). An experimental plan of the trials is shown in Table 10. Culture media were BG11 and F, as in the laboratory screening. BG11 for outdoor cultures was prepared from tap water which was filtered through 10 and 1 μm polypropylene filters (Domnick Hunter, St Neots, UK) and then added with sterile nutrient solutions. F medium was prepared with tap water as described above and treated as for BG11 preparation. In both media nutrient concentration was adjusted as for laboratory trials. For all the trials the initial cell concentration was 0.4 g L^−1^. The first outdoors trial (Table 10, trial 6), was performed in July to test productivity at high temperatures using 1 L, 60 mm in diameter bubble tubes immersed in a water bath, without temperature regulation, in batch regime. The trial was carried out in both BG11 and F media.

The following outdoor trials (Table 10, trials 7 and 8) were carried in GWP-II photobioreactors. These culture systems are made of a flexible low-density polyethylene culture chamber contained within a simple structure comprising a wooden base and a number of vertical metal uprights driven directly into the base [69,75]. The four GWP-II panels used in the experiments were placed vertically, facing north-south, and side by side in a single row. Each panel was 50 cm wide and 70 cm high. The culture was 50 cm high, 10 L in volume and had a surface exposed to direct radiation of 0.25 m^2^. For mixing, compressed air was bubbled at the bottom through a perforated plastic tube; the air-flow rate was 0.5 L L^−1^ min^−1^. CO_2_ was injected with gas diffusers via a valve regulated by a pH control system set at a value of 7.5. The first trials in the GWP-II (Table 10, trial 7), in batch regime, were performed in July - August, to test growth at high temperatures on the isolation medium of each strain or in parallel in BG11 and F medium; cooling was not activated. The second trial (5-day long) in the GWP-II (Table 10, trial 8) was carried out in September to test the culture behaviour under nitrogen starvation, in F medium, in batch regime. In this case the culture was cooled by circulating in a serpentine placed in the reactor cold water when the culture temperature exceeded 40°C. GWP reactors were inoculated with cultures from 1 L bubble tubes kept outdoors in both trials 7 and 8.

### Analytical methods

Culture growth was estimated by biomass dry weight determination: an aliquot of the culture (5 mL) was diluted to 50 mL and filtered on 47 mm membranes with nominal porosity of 1.2 μm (FILTER-LAB, Barcelona, Spain), which were then washed with deionized water (30 mL) and dried at 105°C until constant weight. For cultures grown in laboratory under light:dark cycles and outdoors, the samples for determination of culture dry weight were collected at the end of the dark period.

For biomass composition analyses, the cultures were harvested by centrifugation and washed twice in NaCl solution at 1 g L^−1^ for cultures in BG11 and at 9 g L^−1^ for cultures in F, and centrifuged again. The pellets were frozen and then lyophilized. The biomasses were analyzed for protein [76], carbohydrate [77] and lipid [78]. Ashes were determined using a muffle furnace on about 10 mg of biomass (dry weight). Nitrogen (N-NO_3_^−^) concentration in the medium of nitrogen starved cultures was determined daily, using the method of Ferree and Shannon [79].

Solar radiation data obtained from the Photovoltaic Geographical Information System (PVGIS) were used for calculations reported in Table 7. The monthly average horizontal radiation (Gh) and the ratio of diffuse (Dh) to global radiation (Dh/Gh) were used to derive beam and diffuse average values on the horizontal for the period considered.

Beam radiation on the south-facing vertical panels was then calculated for each month following Kreith and Kreider [80]. Losses of beam radiation due to mutual shading were avoided as distance between panels was optimized for each month. Losses of diffuse radiation, with respect to an isolated reactor, which are a function of the slope of the reactor (β) and the distance between panels (D), were calculated for each month considering a view factor (Fd), representing the fraction of the sky dome viewed by vertical panels placed in parallel rows at a certain distance. A tilt factor for ground-reflected radiation (Rr), taking into account the ground area non-shaded by the panels, was finally calculated to account for the ground-reflected radiation intercepted by the vertical panels [80].

## Abbreviations

β: Slope of the reactor; D: Minimum distance between rows that avoids shading of direct radiation among the panels; Dh: Diffuse radiation on the horizontal; EPA: Eicosapentaenoic acid; Fd: View factor representing the fraction of the sky dome viewed by vertical panels placed in parallel rows at a certain distance; Gh: Global radiation on the horizontal; GWP: Green Wall Panel; PE: Photosynthetic efficiency; PVGIS: Photovoltaic geographical information system; Rr: Ground-reflected radiation.

## Competing interests

The authors declare that they have no competing interests.

## Authors’ contributions

AG carried out the cultivation of the strains, participated in the study design and drafted the manuscript. NBi participated in the study design and in the writing of the manuscript. GS participated in the cultivation of the strains and helped to draft the manuscript. LR participated in the study design and in the writing of the manuscript. NBa participated in the calculation of the productivity at 1 ha scale and in the writing of the manuscript. MRT conceived the study, coordinated and participated in the study design and in the writing of the manuscript. All authors read and approved the final manuscript.
